# Prefrontal-hippocampal interactions supporting the extinction of emotional memories: the retrieval stopping model

**DOI:** 10.1038/s41386-021-01131-1

**Published:** 2021-08-26

**Authors:** Michael C. Anderson, Stan B. Floresco

**Affiliations:** 1 MRC Cognition & Brain Sciences Unit, University of Cambridge, Cambridge, UK; 2 Behavioural and Clinical Neuroscience Institute, University of Cambridge, Cambridge, UK; 3 Department of Psychology, and Djavad Mowafaghian Centre for Brain Health, University of British Columbia, Vancouver, Canada

**Keywords:** Human behaviour, Neuroscience

## Abstract

Neuroimaging has revealed robust interactions between the prefrontal cortex and the hippocampus when people stop memory retrieval. Efforts to stop retrieval can arise when people encounter reminders to unpleasant thoughts they prefer not to think about. Retrieval stopping suppresses hippocampal and amygdala activity, especially when cues elicit aversive memory intrusions, via a broad inhibitory control capacity enabling prepotent response suppression. Repeated retrieval stopping reduces intrusions of unpleasant memories and diminishes their affective tone, outcomes resembling those achieved by the extinction of conditioned emotional responses. Despite this resemblance, the role of inhibitory fronto-hippocampal interactions and retrieval stopping broadly in extinction has received little attention. Here we integrate human and animal research on extinction and retrieval stopping. We argue that reconceptualising extinction to integrate mnemonic inhibitory control with learning would yield a greater understanding of extinction’s relevance to mental health. We hypothesize that fear extinction spontaneously engages retrieval stopping across species, and that controlled suppression of hippocampal and amygdala activity by the prefrontal cortex reduces fearful thoughts. Moreover, we argue that retrieval stopping recruits extinction circuitry to achieve affect regulation, linking extinction to how humans cope with intrusive thoughts. We discuss novel hypotheses derived from this theoretical synthesis.

## Introduction

Thoughts and emotions, like actions, often need to be controlled. For example, when reminded of an unpleasant event, people often try to reduce distress by limiting awareness of the unwanted memory. Attempts to limit awareness are often achieved by retrieval stopping, an intentional effort to terminate the retrieval of content elicited by the reminder. Over the last two decades, evidence has mounted that the prefrontal cortex plays a key role in retrieval stopping. Retrieval stopping recruits a prefrontally-mediated inhibitory control mechanism that, over repeated exposures to reminders, suppresses unwanted traces, rendering them less intrusive, less unpleasant, and less likely to be retrieved [1–3]. Retrieval stopping resembles action stopping, with both engaging right dorsolateral and ventrolateral prefrontal cortices [4–6]. During retrieval stopping, however, instead of modulating motor cortex, the prefrontal cortex targets memory-related regions, suppressing hippocampal and neocortical activity [6–9]. The shared reliance of action and retrieval stopping on lateral prefrontal cortex suggests a domain-general mechanism capable of inhibiting diverse content by dynamically changing its connectivity to differing target areas that require control [5, 6]. Importantly, when suppressing retrieval of unpleasant memories, right lateral prefrontal cortex modulates the hippocampus and amygdala in parallel, altering longer-term affective responses [7, 8], suggesting memory control contributes to emotion regulation [9].

In the last two decades, knowledge about the neural mechanisms of fear extinction also has rapidly expanded [10–13]. Extinction refers to reduced conditioned fear responding that occurs with repeated unreinforced presentations of a conditioned fear stimulus. Although retrieval stopping and extinction are laboratory models relevant to emotion regulation [9], relationships between the phenomena have not been considered. Indeed, extinction historically has been viewed in terms of associative learning theory, not cognitive control. Here we argue that prefrontal inhibitory control mechanisms contribute to extinction through retrieval stopping. First, we elaborate this retrieval stopping hypothesis, highlighting parallels between extinction, retrieval stopping and action cancellation. Next, we describe the functional and anatomical characteristics of retrieval stopping, focusing on prefronto-hippocampal and prefronto-amygdalar interactions. We then review animal and human evidence suggesting that retrieval stopping contributes to extinction. Given these considerations, we propose that fronto-hippocampal interactions during retrieval stopping arise during extinction. We argue that conceptualizing extinction as retrieval stopping introduces a broader perspective that aligns with Ledoux and Pine’s [14] two-system model of fear and anxiety, and that compellingly links extinction to clinically relevant human behavior.

## The retrieval stopping hypothesis of fear extinction

Our central thesis asserts that extinction recruits prefrontally-mediated inhibitory control mechanisms to suppress retrieval, contributing to the affective adaptations normally observed. Consider an example of retrieval suppression, reported by an acquaintance of the first author. One afternoon, the acquaintance was driving down a pleasant residential street when a boy ran out from behind a bush to retrieve a ball before it rolled into the road. Panicking, the friend nearly wrecked his car, even though there was no danger of hitting the boy. He overreacted because the boy’s abrupt appearance reminded him of roadside bomb attacks from his time in the Iraq war. In the ensuing seconds, he suppressed the unpleasant reminding, enabling him to calm down and resume driving. After being home from Iraq for several months, situations like this rarely reminded him of roadside attacks. This illustrates how cues can elicit unwelcome memories automatically, and how important it is to have the ability to stop such intrusions in the immediate and longer-term.

This incident illustrates the kind of example researchers have in mind when arguing for extinction’s relevance to post-traumatic stress disorder (PTSD). Yet, a key aspect of the person’s coping behavior that goes unacknowledged in the extinction analogy is their effort to control retrieval of the intrusive thought through retrieval stopping. The person not only needed to adjust their emotional state, but also how their memories responded to environmental cues, a situation referred to as a memory adaptation problem [3]. If people spontaneously choose to suppress retrieval in such cases, it raises questions about how suppression relates to extinction. According to the retrieval stopping model, retrieval stopping plays a causal role in reducing fear in the standard fear extinction situation; thus, adjusting emotional responses to cues, requires, in part, that organisms solve a memory adaptation problem.

**Fig. 1:**
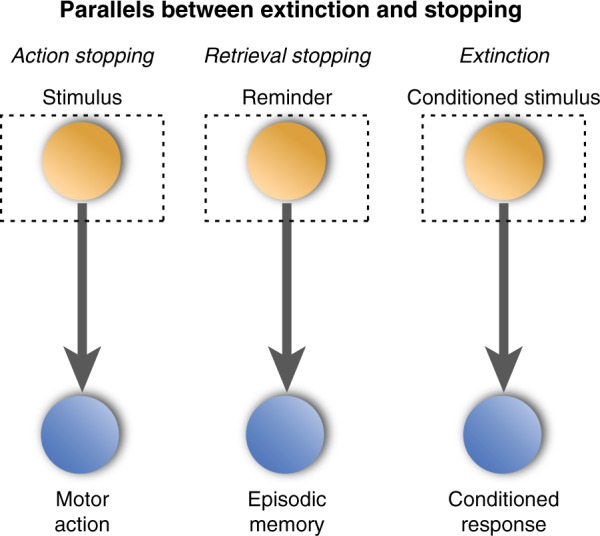
Functional similarities between action stopping, retrieval stopping, and extinction. In each case, an attended stimulus enters working memory (dotted box), driving automatic retrieval of an associated representation (either a motoric representation, an episodic memory or a conditioned emotional response), which the organism stops.

To illustrate the analogy, consider the structural similarities between action and retrieval stopping on the one hand, and extinction on the other (Fig. 1). During action stopping, a person attends to a stimulus that triggers a prepotent action associated to it, that, while usually appropriate, must be stopped. For example, upon seeing an object fall, one might start to catch it reflexively, but realize that it is a potted cactus, which would be unpleasant to catch. People and other organisms can stop actions under such circumstances, an ability mediated by response inhibition [15, 16]. Similarly, during retrieval stopping, a person attends to a reminder that triggers retrieval of a memory that, for various reasons, is unwanted. For example, some reminders may elicit memories of embarrassment, pain, anger, sadness, or guilt, for which most people would be motivated to limit thoughts. Even for neutral thoughts, one may wish to avoid distraction; here too, people can terminate retrieval, limiting awareness of the memory, an ability that may be enabled by mechanisms shared with action stopping [5, 6].

The extinction of conditioned fear resembles action and retrieval stopping. On each trial, the person attends to a stimulus that has become associated to a threat (the conditioned stimulus), leading them to experience fear, and make defensive responses (e.g., freezing). On initial trials, these responses are appropriate, given the prior pairing of the conditioned and unconditioned (aversive) stimuli. But repeated exposures without the unconditioned stimulus make it clear that the conditioned stimulus no longer predicts threat. Correspondingly, the fear reaction ceases to be useful and so it declines. The formal similarity to stopping is unmistakable: in extinction learning, as with action and retrieval stopping, repeated exposures to a cue reduce its tendency to elicit the associated response, under conditions designed to communicate that the response is not needed. Extinction differs from stopping, however, in the presumed intentionality of the process reducing the response. Whereas retrieval and action stopping are accompanied by an explicit goal to terminate retrieval or action production, in extinction, no stopping instruction occurs; rather, any decision about the wisdom of stopping the emotional response, if it occurs, is left to the individual, based on their detection, over exposures to the stimulus, of a decrease in the likelihood of threat.

Some differences among these three situations are more apparent than real. For example, because extinction training provides no overt stopping cue, declines in emotional responding with repetition are easily construed as reflecting an automatic learning process that incrementally adjusts the cue-response association. This interpretation may seem desirable because extinction arises in animals, for which many might be reticent to attribute intentionality; indeed, extinction in invertebrates indicates that some cases of extinction may reflect automatic processes. But all manifestations of extinction need not be painted with the same brush, simply because stopping is not signaled by the task. Absence of stopping cues is a poor reason to assume intentional stopping does not occur. Overt stopping cues rarely exist when action or retrieval stopping occurs “in the real world”; rather, the impetus to stop comes from changes detected by the organism. Stopping oneself from catching a hot pot is initiated by perceptual feedback indicating a threatening outcome; and stopping retrieval of an unwelcome thought begins when we detect a memory or thought that threatens our emotional state. Gradual declines in either conditioned responding or intrusive thoughts may reflect increasing certainty that these responses are no longer relevant and greater willingness to stop them. Under natural conditions, then, extinction and stopping do not intrinsically differ in overt cuing or their apparent intentionality.

Extinction also appears to differ in the content that is controlled, with a strong focus on fear and threat responses. Here again, these differences are more apparent than real. For example, as the example of the Iraq war veteran suggests, episodic memories for conditioning experiences may accompany affective learning and be elicited by conditioned stimuli, at least for mammals with intact hippocampi. Indeed, in human participants, fear conditioning can lead people to develop intrusive images as conditioned responses [17–19], consistent with cognitive theories of PTSD [20]. Actions triggered by the threat response also may become associated to conditioned stimuli. Thus, when animals or humans undergo conditioning, the responses elicited by the cue may include affect, memories and actions; correspondingly, gradually extinguishing conditioned responding may entail reducing fear, actions, and memories, consistent with a memory adaptation problem. Similarly, during retrieval suppression, retrieval cues may elicit episodic memories of aversive images, and emotional responses; and evidence suggests that the hippocampus, amygdala, and neocortical regions are parallel targets of control. Thus, extinction and retrieval stopping appear not to differ intrinsically in the content affected.

Given the foregoing parallels, it becomes important to consider whether retrieval suppression contributes to extinguishing conditioned fear. In our retrieval stopping model, we introduce several assumptions that bridge these phenomena. First, we assume that organisms find fear aversive for several reasons. Fear responses (e.g., behavioral arrest, avoidance) can interfere with rewarding or survival-relevant pursuits such as seeking food, shelter, or mates. Fear states also undermine or bias high-level processes, such as planning, decision making, and behavioral control that are important for pursuing goals [21, 22]. Finally, sustained fear entails arousal and stress, which have energetic costs. Consequently, a natural affect reduction motive can lead to reduced fear responses when they lose relevance, that enable organisms to pursue other goals (Affect reduction motive). Second, we assume that when fear is perceived as unnecessary, the affect reduction motive will trigger prefrontal inhibitory control processes to alter the fear state (Active control assumption). With this assumption, we underscore how extinction, in mammals at least, may entail additional processes that require effort, and are thus not obligatory or context-independent in how effectively they function. How much effort is required is an empirical question [23, 24]. Third, we assume that conditioned stimuli elicit an integrated complex of knowledge associated with the conditioning experience, potentially including affective responses, episodic memories, and actions, and that efforts to regulate fear entail parallel modulation of all associated content, by default (Mnemonic interdependence and Parallel modulation). Modulating affective and mnemonic content in parallel allows extinction to alter both threat responding and subjective fear, which may rely partially on mnemonic traces [14]. Fourth, we assume that the inhibitory mechanism achieving extinction originates from a common prefrontal source, but influences memory, affect, and action via distinct pathways (Mechanistic generality). Finally, we assume that inhibitory control over the integrated conditioning complex can initialize early during extinction learning. However, it takes longer for safety to be perceived and retrieval stopping to begin in early trials. Indeed, fear responses in rodents often actually show a slight increase during the first few trials of extinction training [25–27], but after additional recurring absences of the US, control is evoked more rapidly, and affective responses grow weaker and more short-lived (Regulatory learning assumption). Together, these assumptions provide a framework for when and why extinction engages retrieval suppression. To understand what suppression would do, we turn to behavioral and neural findings about retrieval stopping and how it is studied.

## Retrieval stopping: core behavioral findings

Behavioral and imaging studies usually measure retrieval stopping with the Think/No-Think paradigm (hereinafter, the TNT paradigm; [1]). This procedure models situations in which we encounter a reminder to a memory we do not wish to think about and try to keep the memory out of mind. To create reminders, participants study cue–target pairs (e.g., word pairs, or picture pairs; e.g., “ordeal roach”) and are then trained to recall the second item (roach) whenever they see the first (ordeal) as a reminder. Participants then enter the think/no-think (TNT) phase, in which they exert control over retrieval. On each trial, a reminder from one of the pairs appears in green or red; when reminders appear in green, participants are to recall the response and keep it in mind during the trial; but for red reminders, participants must attend the reminder but suppress retrieval. Usually, a given item is only suppressed or retrieved (not both) and is repeated 8–16 times, much like occurs in extinction experiments. Unlike with extinction, the instruction on no-think trials explicitly asks participants to override retrieval and prevent the associated memory from entering awareness, despite the cue’s tendency to elicit that memory. Participants are told that if the memory comes to mind, they are to suppress it, much like the veteran did in our earlier example. The key question concerns whether people can recruit inhibition to overcome memory intrusions and whether doing so disrupts retention of the excluded memory. To measure suppression’s mnemonic aftereffects, participants complete a test in which they receive each reminder and try to recall the paired memory. Test performance is compared between previously suppressed items (No-think trials), retrieved items (Think trials) and studied items that they neither suppressed nor retrieved during the TNT phase (Baseline trials). Comparing recall of No-Think items to Think or Baseline items indicates whether suppression affected retention.

**Fig. 2:**
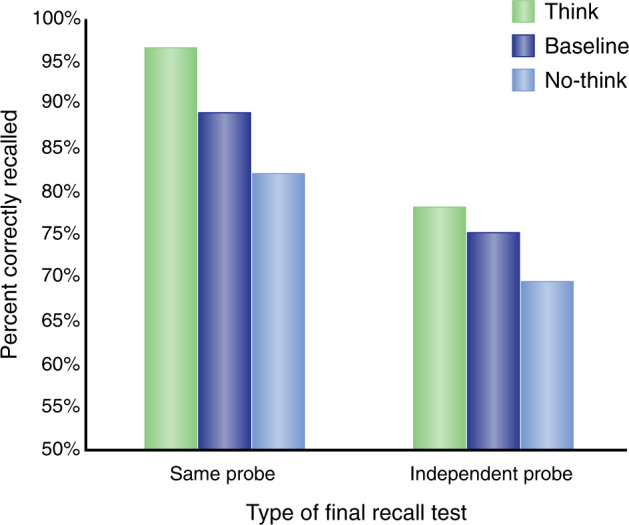
Final recall performance in the Think/No-Think procedure, aggregating over 687 participants in many different studies [154]. The graph shows the percentage of items recalled as a function of whether people thought of the item (think), suppressed the item (no-think), or had no reminders to the item during the think/no-think phase (baseline). Left side: Recall when tested with the originally trained reminder (i.e., the same probe). Right side: Recall when tested with a novel reminder, such as a category name for the suppressed response.

Research using the TNT procedure shows that people can stop retrieval. Several notable effects support this conclusion. First, retrieval stopping abolishes the benefits of reminders on memory, as reflected by often large differences between Think and No-Think items on delayed tests (Fig. 2). Indeed, reminders to No-Think items can be repeated over a dozen times with little benefit in later recall of the associated traces. Thus, at a minimum, stopping retrieval reduces the facilitation that reminders usually promote, limiting integration of unwanted experiences into long-term memory. Second, suppressing retrieval often reduces No-Think item recall below that observed for Baseline items, a phenomenon known as suppression-induced forgetting (SIF). SIF indicates that during retrieval stopping, reminders trigger mechanisms that impair access to the memory. Third, SIF occurs even when the suppressed item is tested with a novel cue, indicating a generalized impairment (right side, Fig. 2). This “cue-independence” suggests that the forgetting arising from retrieval stopping is not primarily associative, as its occurrence often does not depend on testing with a particular cue [1] (though associative components can contribute—see [28]). Most of these effects have been observed with both verbal and visual cue–target pairs (e.g., face–scene associations), and the effects arise for emotionally unpleasant target items [2, 3, 29]. SIF has even been observed with autobiographical memories, although primarily in memory for details [30]. Thus, stopping retrieval is achieved in part by suppressing the associated memory, consistent with inhibitory control. Revealingly, this inhibitory process is coarsely targeted: stopping retrieval not only harms memory for items linked to the reminder, but also entirely unrelated events occurring before and after the retrieval stopping trial [31, 32]. This remarkable amnesic shadow suggests that retrieval stopping globally disrupts hippocampal state, yielding anterograde and retrograde amnesia for other hippocampal traces.

Beyond its effects on explicit memory, repeatedly presenting a cue and stopping retrieval gradually reduces the memory’s tendency to intrude and people’s affective responses to the suppressed content. Because intrusions are, by nature, involuntary retrievals yielding no observable behavior, they must be probed with self-reports. For example, many studies identify intrusions by asking, after each No-Think trial, whether the cue elicited the memory, despite participants’ efforts to stop retrieval. Studies often collect these reports during an fMRI or EEG protocol to determine whether they identify distinct neural mechanisms of the intrusion response (see the “Retrieval stopping: neural mechanisms” section). Remarkably, whereas participants commonly experience involuntary intrusions on early trials (often around 60% of trials), their incidence declines robustly after repeated stopping, showing proportional reductions of nearly 50% [8, 33–38]. This gradual decline echoes reductions in conditioned responding during extinction. Interestingly, intrusion declines often predict later SIF effects in explicit memory, pointing to a common mechanism contributing to these effects [33, 38]. These findings suggest that engaging inhibitory control to suppress intrusions ought to affect spontaneous retrieval in daily life, a possibility consistent with large SIF effects on free association tests [39]. Indeed, SIF broadly affects implicit memory, in which retrieval is unintentional [40]. Stopping retrieval reduces later indirect expressions of the suppressed content on perceptually-driven tests such as perceptual identification [41, 42] and conceptually-driven tasks that measure accessibility of ideas underlying the suppressed content [39, 43–45].

## Retrieval stopping: neural mechanisms

fMRI studies have revealed contributions of the prefrontal cortex to retrieval stopping, and how this region modulates brain activity relating to memory and emotion. Here we summarize which prefrontal regions suppression engages, and under what conditions, and evidence for how it modulates the hippocampus and the amygdala.

### Prefrontal regions involved in stopping retrieval

To isolate prefrontal regions involved in retrieval stopping, neuroimaging studies have scanned participants as they performed the Think/No-Think task. All trials in this task present a cue from a trained pair. Some trials ask participants to retrieve the paired item (Think trials), whereas others cue them to stop retrieval (No-Think trials), typically signaled by green or red colored task cues, respectively. Regions more activated during No-Think than Think trials reflect recruitment of stopping-related processes above and beyond processes involved in cue processing and retrieval, as reflected in increased blood-oxygenation-level-dependent signal (BOLD signal). Retrieval stopping studies have examined word pairs, face-scene pairs, word-scene pairs, word-face, and word-object pairings of both neutral and negative valence [2, 3, 29]. Particularly relevant to extinction, retrieval stopping has been examined for person-specific fears about the future [46].

Retrieval stopping engages right-lateralized prefrontal regions, including dorsolateral prefrontal cortex (DLPFC), ventrolateral prefrontal cortex (VLPFC), posterior middle frontal gyrus (pMFG), and bilateral insula. Amongst these, the most extensive activations arise in right DLPFC, which often extend the anterior–posterior length of the MFG, in a region spanning Brodmann’s areas (BA) 9 and 46. Anteriorly, the right DLPFC activation usually extends into the posterior aspect of BA10, bordering 9/46. Indeed, DLPFC activations sometimes are restricted to this anterior BA9/46/10 area, suggesting it is a key DLPFC region of retrieval stopping [4].

Several factors have led us to hypothesize that anterior DLPFC (aDLPFC) implements a top-down inhibitory control signal that suppresses hippocampal processing, interrupting retrieval. First, right aDLPFC is particularly engaged when overriding retrieval, and not during other strategies for preventing a memory from coming to mind. For example, an alternative approach would involve retrieving substitute thoughts that pre-empt or supplant the to-be-avoided memory in awareness. Controlling unwelcome memories by thought substitution, however, engages left VLPFC regions involved in retrieval [47]. These left VLPFC regions resemble those involved in cognitive reappraisal of emotion, raising the possibility that reappraisal builds on substitution mechanisms [9]. In contrast, instructing participants to eschew thought substitutes, and instead focus on stopping retrieval, engages right aDLPFC and VLPFC, but not left prefrontal cortex, suggesting that the former regions contribute to retrieval stopping. Consistent with this, right aDLPFC is more engaged when a cue elicits an intrusion that needs to be purged, compared to when it does not [34]. Finally, as we will discuss, effective connectivity analyses reveal that right aDLPFC negatively modulates hippocampal activity during retrieval stopping. The function of right pMFG remains unknown.

Evidence suggests that the right aDLPFC and VLPFC regions engaged during retrieval stopping also contribute to action stopping. For example, Apsvalka et al. [6] found common activations in right aDLPFC and VLPFC across action and retrieval stopping tasks within participants, with the regions strongly agreeing with those revealed in a quantitative meta-analysis of action and retrieval stopping (Fig. 2). Both regions predicted forgetting of suppressed thoughts and more efficient action stopping. Apsvalka et al. found that aDLPFC and VLPFC acted in concert, switching their effective connectivity between the hippocampus and motor cortex when changing from retrieval stopping to action stopping blocks, as would be expected from a flexibly targeted inhibition process. Consistent with a domain-general mechanism, training a pattern classifier to distinguish activity patterns in aDLPFC and VLPFC relating to Action Stopping versus Going enabled it to discern whether people were suppressing their thoughts in the TNT task. The prefrontal regions showing this domain-general pattern were a subset of the regions involved in retrieval stopping more broadly, especially right aDLPFC (BA 9/10/46) and VLPFC (BA44/45) (Fig. 2). Converging evidence for the causal necessity comes from patients with lesions to the right LPFC, who show no SIF, in contrast to patients with left LPFC lesions [48], echoing similar lateralization in motor stopping [15]. Thus, the similarity between action and retrieval stopping in Fig. 1 may be more than a metaphor and may be realized in the brain by a common stopping mechanism (see also [49]). Critically, this aDLPFC region also is engaged when people seek to stop retrieval of emotional memories [7, 8], consistent with a potential role in fear extinction. The role of right aDLPFC and VLPFC in stopping across these diverse domains positions it to stop the complex of affective, episodic, and motoric responses triggered by conditioned stimuli, consistent with the retrieval stopping model.

**Fig. 3:**
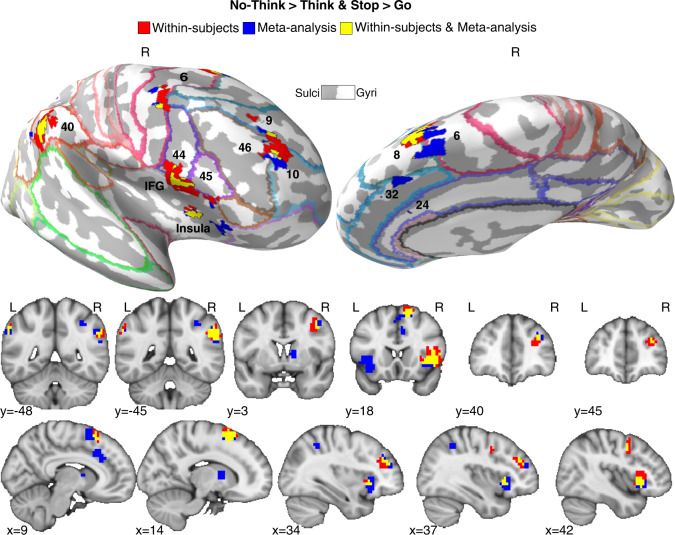
Brain regions engaged during both action stopping and retrieval stopping. Red areas reflect the activations arising after a within-subjects conjunction (Stop action > Go & No-Think > Think) for people asked to stop actions and stop retrieval; blue areas reflect the results of a meta-analytic conjunction across 56 separate studies of action stopping (*N* = 40) and retrieval stopping (*N* = 16); yellow areas reflect the overlap between the meta-analytic and within-subjects conjunctions. Demarcated regions are Brodmann’s areas. Key clusters of activation include the right anterior DLPFC (BA 9/46/10), the right inferior frontal gyrus (IFG, BA44/45) and Insula and ACC (BA32). Apart from the Insula, relatively few left-sided activations arise, and so are not depicted.

Retrieval and action stopping also both engage frontal midline areas (Fig. 3), including the anterior cingulate cortex (ACC) and the pre-supplementary motor area (preSMA). Within the ACC, the most consistently engaged region is BA32, though smaller activations in BA24 occur. We cannot know from this shared anatomy whether these regions serve the same function in retrieval stopping and action stopping, nor do we know what that function is. One possible mechanism would involve detecting conflict created by an unwelcome intrusion and also in mediating the influence of the LPFC on the hippocampus [50]. Recent work suggests that the shared activations during action and retrieval stopping reflect multiple networks, dominated by the Cingulo-Opercular and Frontal-Parietal networks, with the aDLPFC contributing to the former [6].

### Prefrontal modulation of the hippocampus, amygdala, and cortex during retrieval stopping

Although retrieval and action stopping engage common prefrontal regions, these regions have different effects across task contexts [6, 51]. Whereas action stopping modulates motor cortical areas via the striatum (e.g., [4, 52, 53]), retrieval suppression reduces BOLD activation in the medial temporal lobes, including the hippocampus and entorhinal and perirhinal cortex [8, 33, 47] via pathways which remain to be identified. Activation during No-Think trials is lower than during Think trials in left and right hippocampi and these suppression-related reductions occur throughout the long-axis of the hippocampus.

Reduced hippocampal BOLD activity on No-Think vs Think trials need not indicate active downregulation and may reflect hippocampal engagement during Think trials. Thus, on No-Think trials, participants may simply fail to engage retrieval. Moreover, the relation between BOLD signal and neural activity is more complex in the hippocampus than in other brain regions, suggesting that reduced BOLD may not always signify reduced neural processing [54]. Evidence has grown, however, that inhibitory control actively nodulates hippocampal activation during retrieval stopping. First, hippocampal activity is reduced compared to activity during a fixation baseline condition [7, 47], suggesting that reductions reflect more than a mere lack of retrieval. Second, DLPFC activation during No-Think trials is often negatively correlated with hippocampal activity [7, 55]. Third, reduced hippocampal activity predicts later SIF [7, 47]. Fourth, effective connectivity analyses using dynamic causal modeling reveal that right aDLPFC modulates the hippocampus [6, 8, 34, 35, 41, 47, 51], with negative coupling from aDLPFC predicting both SIF [47] and reduced intrusions over blocks [34]. Right aDLPFC appears to act in concert with right VLPFC in exerting this influence, just as they appear to work in concert to influence motor cortex during action stopping [6]. Converging evidence comes from intracranial recordings in humans, which reveal effective connectivity between the DLPFC and the hippocampus in the beta range, during intentional forgetting [56].

Importantly, hippocampal GABA plays an anatomically specific role in enabling the aDLPFC to regulate hippocampal activity. Participants with higher concentrations of hippocampal (but not prefrontal or visual cortical) GABA measured with single proton MRS, show greater downregulation during retrieval stopping, more successful forgetting of intruding thoughts, and greater negative coupling between the right aDLPFC and the hippocampus [51] than do participants with lower GABA concentrations. As such, it has been hypothesized that hippocampal GABA provides a pivotal function that enables the aDLPFC to implement a long-range inhibitory influence needed to control intrusive thoughts. These findings may indicate the importance of inhibitory interneuron populations within the hippocampus in stopping retrieval and disrupting the hippocampal representation of the suppressed event. GABA-induced disruptions to hippocampal function may contribute to the amnesic shadow, taken to reflect dysfunctional hippocampal encoding and stabilization caused by retrieval stopping [31, 32].

**Fig. 4:**
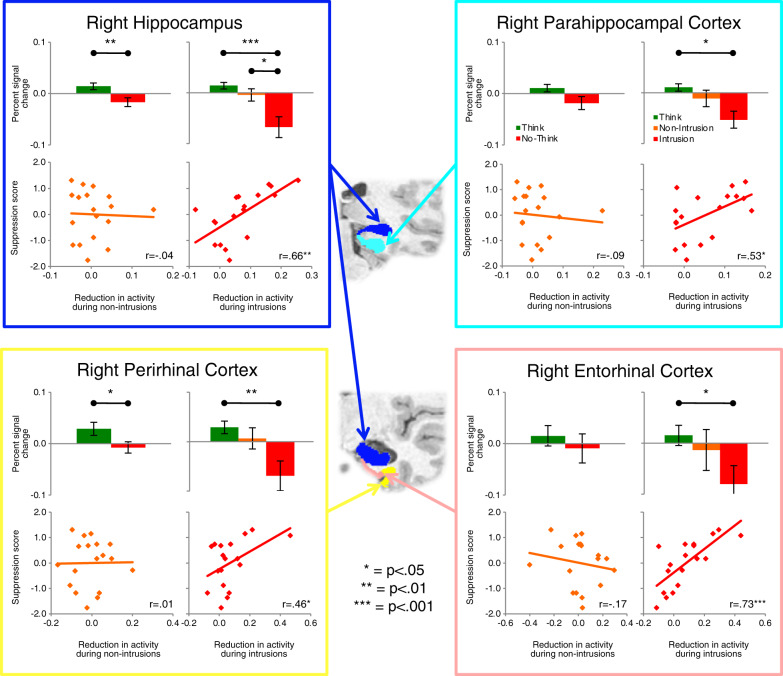
Activation in medial temporal lobe ROIs and its relationship to suppression scores. Each quadrant represents an ROI (illustrated in the coronal slices in the center of the figure) and within those quadrants are four panels: activity during Think (green bar) and No-Think (red bar) trials, without distinguishing between intrusions and non-intrusions (upper left); activity during Think (green bar), non-intrusion (orange bar), and intrusion trials (red bar, upper right); the relationship between behavioral suppression scores and downregulation of this region during non-intrusion trials (lower left); and the same relationships during intrusion trials (lower right). In scatterplots, behavioral (memory) suppression scores are expressed in z-units, with higher values indicating more forgetting; downregulation of hippocampal activity (*x* axis) is expressed as a positive value). The value of distinguishing intrusions is reflected in the greater suppression-related downregulation during intrusions throughout the panels, and in its robust prediction of forgetting. All error bars reflect SEM.

Evidence suggests that intrusions during retrieval stopping contribute to triggering prefrontal control over hippocampal activity. Studies using intrusion reports illustrate this point [8, 33, 35]. For example, we isolated No-Think trials on which an unwanted memory entered a participant’s awareness using trial-by-trial intrusion reports and linked these reports to hippocampal activity [33]. Although modest hippocampal downregulation occurred on non-intrusion trials, intrusions were accompanied by pronounced reductions (Fig. 4), consistent with the purging of the recollection from awareness. Strikingly, the deeper the downregulation during intrusions, the more SIF people later exhibited (*r* = 0.66). Non-intrusions did not reveal such a correlation. Intrusion-related downregulations also were associated with more spatially extensive modulation of medial temporal lobe regions, including anterior and posterior hippocampus, entorhinal, perirhinal, and parahippocampal cortices (Fig. 4). Relating this to the Iraq War veteran discussed at the outset, perhaps their intrusion of an upsetting event precipitated a robust retrieval stopping response that broadly impacted the medial temporal lobes, disrupting memory.

**Fig. 5:**
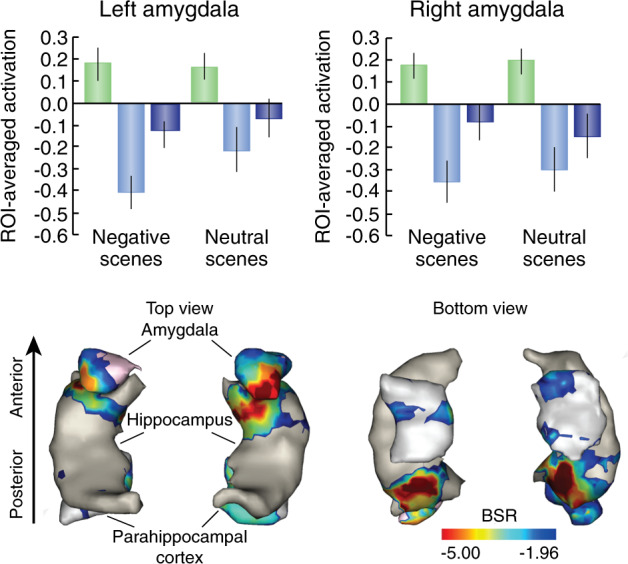
The suppression of amygdala activity during retrieval stopping of aversive scenes and its relation to affect regulation. Top Panels. Whereas retrieving an aversive image during Think trials increased left and right amygdala activity (Green bars), suppressing retrieval on No-Think trials reduced it, more so during intrusions (light blue bars) than non-intrusions (blue bars). A similar though weaker pattern arose for neutral scenes. Bottom Panels. Intrusion-related down-regulations in the amygdala and anterior hippocampus were related to both reduced intrusion frequency and affect suppression (a reduction in perceived negative valence for the suppressed content), according to a behavioral partial least squares (PLS) analysis. Represented here are those voxels significantly associated to the first significant latent variable from PLS, whose downregulation significantly correlated with intrusion proportion for both Negative and Neutral scenes, as well as with affect suppression exclusively for Negative scenes. Error bars indicate bootstrapped 95% CI.

Disrupting episodic memory may not help if suppression leaves affective learning associated with the unpleasant experience unaffected. Several studies have now found that stopping retrieval reduces amygdala activity bilaterally when suppressing aversive scenes [7, 8, 55, 57], unpleasant words [58], or even participant-specific fears of the future [46]. Paralleling the hippocampus, suppression-related reductions in amygdala activity are especially pronounced when the aversive image intrudes into awareness and needs to be purged (Fig. 5, [8]). Amygdala downregulation during memory intrusions predicts two important behavioral impacts on the aversive image: reduced intrusion frequency and diminished perception of negative valence in the scene, when later confronted with it. These effects suggest persisting aftereffects of prefrontal control on the amygdala and its affective representations. Consistent with this possibility, retrieval stopping reduces physiological responses to scenes after suppression [37, 57, 59]. These affective attenuation effects likely reflect modulation by right LPFC. Effective connectivity analyses indicate that the right DLPFC modulates the amygdala and hippocampus (especially anterior hippocampus) in parallel as an integrated stopping response to the unpleasant scene and that this modulation is inhibitory, especially during intrusions [8] (Fig. 3). The pathway through which the aDLPFC and VLPFC modulate amygdala activity has not been established, nor has the localization of the suppression within the amygdala.

Beyond the hippocampus and the amygdala, evidence suggests that inhibitory control also targets cortical representations during retrieval stopping. For example, when a cue elicits an image of a visual object, suppression downregulates the hippocampus, but also regions such as the fusiform cortex, involved in the conscious perception of objects [41]. Strikingly, suppressing fusiform cortex induces persisting changes measurable on later tests that engage this region: suppressed objects show reduced neural priming in fusiform cortex compared to unsuppressed objects on perceptual identification tests [41]. This pattern suggests that retrieval stopping disrupts cortical traces needed to perceive or retrieve the object. Similarly, suppressing scenes downregulates the parahippocampal place area, together with the hippocampus. Such findings have prompted the generalization that suppression affects those areas outside the hippocampus involved in reinstating the unwanted memory in parallel with the hippocampus, a pattern known as the reinstatement principle [8, 41, 45]. The impact of suppression on cortical representations provides a mechanism by which it could alter subjective affect perception, which may rely not only on the amygdala, but also cortical representations [14].

### Pathways mediating the influence of the prefrontal cortex on hippocampal activity

**Fig. 6:**
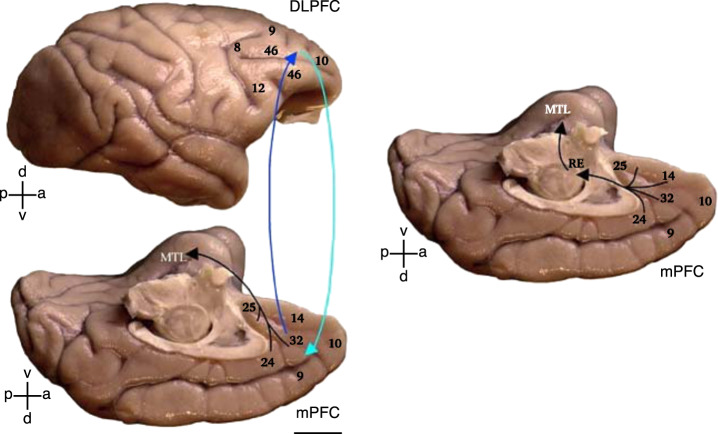
Pathways linking the lateral and medial prefrontal cortices with the medial temporal lobe (MTL) memory system. Top: Lateral surface of the rhesus monkey (*Macaca mulatta*) brain shows the location of Brodmann’s areas 9 (lateral), 46, frontopolar area 10 and areas 8 and 12. Bottom: Medial surface shows the medial extent of areas 9 and 10, cingulate areas 24 and 32, and ventromedial areas 14 and 25. Lateral and medial prefrontal areas have robust bidirectional connections (cyan and blue arrows). The hypothesized entorhinal gating mechanism builds on the predominant projection to the medial temporal lobe (MTL), which originates from the medial prefrontal areas in the anterior cingulate and terminates in the entorhinal (area 28) and perirhinal (area 35) cortices. Right: The thalamo-hippocampal suppression hypothesis builds on the ACC-thalamic nucleus reuniens (RE) pathway. The medial prefrontal cortex (mPFC) areas that receive robust projections from the hippocampus (HPC) (in the anterior cingulate cortex region) send a pathway to the RE that, in turn, originates one of the most prominent thalamic pathways to the medial temporal lobe (MTL), which terminates in CA1 as well as the subicular and rhinal cortices.

The pathways enabling the right aDLPFC and VLPFC to suppress hippocampal activity remain to be established. We have discussed several possibilities based on primate and rodent anatomical studies [50]. We developed a dual pathway hypothesis for how the right DLPFC could stop retrieval either proactively and reactively (Fig. 6). On the one hand, preventing retrieval, given an unwelcome reminder (proactive control) may arise via entorhinal gating (left side, Fig. 6). By this hypothesis, the right DLPFC acts, via the ACC (BA32), to suppress hippocampal inputs, preventing cue information from triggering hippocampal pattern completion. Gating may arise via excitatory projections from BA32 that drive interneurons in the deep layers of the entorhinal cortex which, through ascending projections to superficial layers, truncate hippocampal inputs. As such, cues do not evoke hippocampal traces, and so memories require no inhibition. When entorhinal gating fails, however, cue input reaches the hippocampus and pattern completion ensues, leading to hippocampal retrieval, and cascading activity from the hippocampus to neocortical regions associated with the original experience. This intrusion is proposed to trigger a reactive control path that interrupts hippocampal activity via GABAergic inhibition (right, Fig. 6). We hypothesized that ACC achieves this hippocampal suppression via the nucleus reuniens in the thalamus, which projects, in part, to inhibitory neurons throughout the hippocampus [50]. Although this dual-process model has not been tested during retrieval stopping, recent work on extinction provides evidence consistent with the reuniens pathway, as discussed shortly.

## Evidence for a role of retrieval stopping during extinction in rodents

Retrieval stopping clearly supports people’s ability to overcome intrusive memories. Might this process contribute to extinction? Here we discuss rodent evidence that support two aspects of our claim: (i) the existence of a domain-general source of inhibitory control over memory, action, and affect; and (ii) the recruitment of this prefrontal region to suppress hippocampal activity related to unwanted traces during extinction, via our hypothesized thalamo-hippocampal pathway [50].

### Infralimbic prefrontal cortex as a domain-general source of inhibitory control

The retrieval stopping model predicts that during extinction learning, rodents should engage inhibitory control mechanisms supported by the prefrontal cortex to modulate amygdala and hippocampal in parallel, suppressing both affective responding and memory for the conditioning event. More broadly, we hypothesized a prefrontal inhibitory control source that inhibits actions, memories, and affect, irrespective of the extinction context. Evaluating this mechanistic generality prediction requires consideration of the functional or anatomical homologies of cortical regions between species. Effective connectivity analyses in humans suggest that the DLPFC and VLPFC may originate inhibitory control signals during action and retrieval stopping, affecting motor cortex, hippocampus and amygdala. In rodents, older theories posited that the medial PFC may be functionally homologous to the primate DLPFC, because lesions to it impair working memory and set shifting, as occurs in patients with DLPFC damage [60, 61]. This idea has been challenged on neuroanatomical and neurophysiological grounds [62, 63] and the consensus is that there may be no direct rodent homolog to the DLPFC. Nevertheless, how the rodent medial PFC regulates the stopping of actions and conditioned responses may functionally resemble how retrieval stopping is accomplished. In turn, this can provide important insights into common principles of operation across different frontal lobe regions. Next, we build the case that infralimbic (IL) PFC acts as a source of inhibitory control over action, emotion and memory in rodents. Based in part on striatal and amygdala connectivity, some have argued that this region may be anatomically homologous to Area 25 in primates [64], although other evidence suggests it is homologous to primate posterior lateral orbital prefrontal cortex [65]. Thus, whether Area 25 is functionally homologous across species remains to be resolved [66].

#### Inhibiting actions

Many subregions of the rodent frontal lobes have been implicated in suppressing actions or conditioned responses under certain conditions. These include the lateral and medial orbitofrontal cortex [67–69] and the medial prelimbic PFC [70–72]. However, the ventromedial IL has received considerable attention for its involvement in suppressing behaviors across a variety of domains. Importantly, the idea that the IL is merely a “brake” on a variety of behaviors has been scrutinized [73, 74] and in some instances, this region may also contribute to the expression of conditioned fear responses and instrumental avoidance [72, 75, 76]. Nevertheless, numerous reports implicate the IL in suppressing actions that are inappropriate, non-rewarded or that may lead to punishment. For example, in rats performing a 5-choice serial reaction time task, animals must wait for a “go” signal before making a response, with premature responses incurring a time-out penalty and reduced opportunities for obtaining rewards. Manipulation of the IL PFC increases premature responding, a measure of motor impulsivity indicative of impairments in “waiting” [77, 78]. Similarly, inactivating the IL increases previously extinguished lever pressing associated with cocaine seeking [79], increases non-reinforced actions on a discriminative stimulus task [80] and impairs inhibitory reward seeking on a go/no-go task when discriminative cues signal that actions should be withheld [72]. Response inhibition in service of avoiding punishment also depends on IL function, as inactivations impair behavioral restraint when rats must withhold from retrieving food during a 12 s threat cue predictive of shock [71]. Similarly, the IL has also been implicated in inhibitory avoidance, as IL inactivation increases responding when external discriminative cues signal that doing so will deliver a shock [72]. The IL also contributes to complex forms of action selection during cost/benefit decision making, suppressing the urge to pursue larger, uncertain rewards when cues signal that they are unlikely to be received [81].

Studies of the broader circuitry underlying IL action suppression have focused on its projections to the basal ganglia, and notably on the nucleus accumbens shell. Inactivating the NAc shell induces disinhibitory effects on action suppression that resemble those induced by perturbations in IL functioning [79, 82–84], as does suppressing IL inputs to the accumbens shell [85, 86].

#### Suppressing conditioned emotional responses

Robust evidence indicates that the IL PFC also supports fear extinction. Pavlovian fear conditioning in rodents involves pairing a conditioned stimulus (CS; a cue light/tone or context) with an aversive outcome, such as foot shock (US). Fear conditioning is measured via a conditioned response (CR; e.g., freezing) to the CS. After conditioning, the CS is repeatedly presented alone in an extinction learning phase. The amount of extinction learning is measured at the end of the extinction training (within-session extinction) or after a delay, often 24 h (extinction recall). Extinction learning and recall resemble the Think/No-Think and Final Test phases in retrieval stopping research. Unlike retrieval stopping, however, extinction procedures usually do not provide explicit signals to suppress a CR; instead, rodents must learn, over repeated exposures of a CS that it no longer predicts aversive events. For these reasons, studies using safety signals may more closely resemble the retrieval stopping tasks. Studies using both approaches, however, yield evidence that IL contributes to suppressing Pavlovian reactions especially toward negatively-valenced associations (studies of its role in suppressing appetitive Pavlovian responses have yielded inconsistent findings—e.g., [87, 88]).

If IL contributes to suppressing conditioned fear, disabling it should impair extinction learning and extinction recall. Consistent with this, inactivating IL impairs within-session extinction learning [89, 90] and the expression of that learning on subsequent recall (as evidenced by greater fear responses to the CS). Permanent lesions induced prior to conditioning produce similar deficits in extinction recall [91]. Conversely, stimulation of IL improves extinction learning [92]. During extinction learning, IL activity may induce plasticity within basolateral amygdala nuclei [93]. During extinction recall trials, IL neurons display increased activity to CS cues, and may thus exert online top-down suppression of conditioned fear responses [10, 94]. This suppression arises via projections from IL to amygdalar regions including the GABAergic intercalated cells that reside between the lateral and central nuclei [95–98].

Focused evidence for IL’s role in inhibiting conditioned fear comes from studies employing safety signals. In one study, animals were required to discriminate between different cues paired with reward, shock or nothing (safety cue). IL inactivations led to more freezing in the presence of the safety cue [99]. Similarly, a more a recent study used a modified fear-potentiated startle procedure, wherein during conditioning, animals received unsignaled shocks, interspersed with a light safety cue that was explicitly unpaired with shock. During the test, presenting the safety cue to control animals reduced their startle response compared to that observed without the cue. Yet, inactivation of the IL during the test (but notably, not during conditioning) abolished this safety cue’s ability to attenuate the fear response [100]. These safety signals resemble instructions, in human studies, that the default emotional response to the situation can be stopped, consistent with a role of inhibitory control in fear regulation.

In addition to suppressing Pavlovian fear responses after extinction or in the presence of safety cues, the IL plays a role in extinguishing instrumental avoidance responses. These studies employed a signaled, platform avoidance procedure, wherein rats pressed a lever for food, but presentation of a threat cue required them to cease reward-seeking and move to a safe location to avoid a shock. After training rats to avoid the threat of shock, extinction was performed on this instrumental avoidance task. Under these conditions, IL inactivation impaired consolidation of the extinction of the avoidance response [101]. Avoidance extinction was associated with increased activation of IL neurons that projected to the nucleus accumbens and basolateral amygdala [102, 103], providing another example of the IL exerting top-down control over subcortical structures to suppress conditioned responses.

As alluded to above, the IL is thought to regulate suppression of Pavlovian fear memories primarily via top-down control over amygdalar and striatal nuclei. According to our retrieval stopping model, however, fear regulation in extinction is not entirely about controlling affective content, but also mnemonic representations in the hippocampus, as has been found in retrieval stopping studies. Although the hippocampus is a major contributor to acquiring and expressing fear extinction, work to date has focused little on whether the IL exerts a top-down modulatory impact on hippocampal activity during extinction learning or recall (inhibitory or otherwise). Instead, work on the hippocampus’s role in extinction in both humans and animals generally has focused on bottom-up signals from either the ventral [104, 105] or dorsal [106] hippocampus and also on its function in representing spatial context. However, as discussed shortly, a handful of preclinical studies have alluded to the possibility that indirect pathways from the IL to the hippocampus may facilitate extinction.

#### Suppressing memory

Evidence that rodent PFC can stop retrieval is more indirect. Unlike with humans, rats cannot be instructed to stop retrieval and prevent an associated memory from entering awareness. However, other functional contexts may engage inhibitory control over memory in a more targeted way, to serve other ends. Relevant to our retrieval stopping model is the fact that the PFC plays a central role in controlling retrieval of memories processed by hippocampus. Although PFC lesions in humans and rodents do not disrupt encoding of episodic or contextual memories, these lesions do impair targeted retrieval of specific memories and increase intrusions of irrelevant memories under interference conditions [107, 108], or when rats must distinguish the source or context of information learned (e.g., reviewed by [109]). It has been proposed that the PFC aids in selecting context-appropriate memories by suppressing retrieval of representations that are irrelevant in a situation. For example, in a context-guided memory retrieval task, rats were trained to discriminate two odors (X and Y). In one context, odor X was rewarded, and in the other context, Y was rewarded. Medial PFC lesions do not impair learning of simple odor discriminations [110], but inactivating this structure markedly impairs performance on the context-guided task, making rats more likely to approach the inappropriate odor for a given context [111].

Neurophysiological evidence also suggests that the rodent medial PFC can suppress hippocampal memories. In rats performing the context-guided memory task described above [111], dorsal hippocampal neurons were found to encode context-relevant odors via selective patterns of firing that occurred when animals approached a specific object in particular places in each context. Thus, a group of neurons would fire when the rat approached odor X in one context, but not the other context. However, inactivating medial PFC caused more nonselective patterns of firing in hippocampal neurons, which was thought to underlie indiscriminate retrieval of both appropriate and inappropriate memories. Navawongse and Eichenbaum argued that this finding suggested that when the medial PFC is intact, it suppresses hippocampal neural ensembles that encode competing memories, the persisting activity of which would interfere with the recall of the more context-appropriate trace.

If the medial PFC suppresses competing memories, suppression’s aftereffects might harm later competitor recall. Indeed, a large volume of human work on retrieval-induced forgetting (RIF) indicates that selective retrieval disrupts the retention of competing memories [3, 29, 112]. Recently, RIF has been shown to arise in rodents and to rely on medial PFC integrity [113]. Bekinschtein et al. adapted the spontaneous object recognition paradigm, wherein recognition of a previously explored object can be detected by measuring an animal’s preference to explore an alternative novel object over the familiar one. In the adapted version, animals were placed in an arena context and explored two objects (A and B), each on a different occasion. They then received three “retrieval practice” sessions, each pitting one of the original objects (A) with different novel ones. Unsurprisingly, in each session, the animals spent more time exploring the novel object, a preference that implies that they had retrieved the prior event of exploring A and elected to instead devote their attention elsewhere. After retrieval practice, a critical test assessed memory for the competing object, B, to see whether repeatedly retrieving A had affected it. Consistent with human work on RIF, final recognition of B was impaired, relative to a control condition in which A & B had both been encoded, but no retrieval practice on A had intervened. These authors showed that impaired recognition for B was not caused by heightened interference from A, but rather by an active inhibition process; this inhibition was abolished by inactivating medial PFC prior to retrieval practice, eliminating RIF. RIF can be either abolished or increased by blocking or enhancing dopamine in medial PFC, or by inactivating the ventral tegmental area, showing that mnemonic inhibition depends on prefrontal dopamine [114]). Similar RIF effects were observed by Wu and colleagues [115] using an odor discrimination procedure. Here, inactivating the medial PFC or hippocampus both impaired RIF, alluding to an interaction between these systems to regulate memory inhibition. Together, these findings suggest that medial PFC suppresses distracting memories, possibly via targeted hippocampal inhibition, impairing those traces. Whether those inhibition processes can stop retrieval unselectively, via global inhibition, remains unknown.

### Infralimbic prefrontal cortex and regulation of the hippocampus via nucleus reuniens

The foregoing discussion illustrates how rodent IL cortex may mediate inhibitory control over multiple domains and suggests that it does so by modulating subcortical systems including amygdalar, striatal, and thalamic nuclei, and the hippocampus. Yet, the pathway mediating IL control over hippocampal activity in rats remains to be addressed. As noted earlier, the influence of IL on hippocampal activity may be achieved indirectly via the nucleus reuinens, which we refer to as the thalamo-hippocampal suppression hypothesis [50]. The medial PFC in rodents does not directly project to the hippocampal formation, but it sends excitatory projections to nucleus reuniens neurons, and some of these cells project to the hippocampal formation [116]. Interestingly, nucleus reuniens projections form asymmetric (excitatory) contacts onto CA1 hippocampal neurons, but a considerable proportion of these terminate on inhibitory GABAergic interneurons [117]. Accordingly, stimulating the nucleus reuniens can evoke inhibitory responses in hippocampal cells [118]. Activating nucleus reuniens neurons via prefrontal inputs, therefore, may be one mechanism that can suppress hippocampal activation, facilitating extinction learning and recall and, suppressing memory for event details.

Partial support for this pathway comes from fear extinction studies. Ramanathan and colleagues [119] revealed that reuniens cells display increased firing during extinction recall, which could suppress hippocampal activity. Accordingly, the authors further showed that inactivation of the nucleus reuniens impairs both extinction learning and recall, consistent with a potential mediating role of this structure in inhibiting hippocampal traces of the conditioning events. Furthermore, the same study delineated a direct link between the PFC and reuniens in mediating extinction, as chemogenetically silencing prefrontal neurons that project to this region impaired extinction memory expression. This study did not clarify whether prelimbic vs IL cortical neurons drove these extinction effects, nor did it establish a role for reuniens-hippocampal circuitry, which remain important topics for future studies. Nevertheless, these findings provide strong initial evidence that this trisynaptic circuit may be a key pathway through which the PFC exerts top-down inhibitory control over the hippocampus to suppress fear memories. If so, the IL-reuniens-hippocampus pathway may enable a process homologous to retrieval stopping in humans.

Although the foregoing findings suggest that reuniens might enable an inhibitory influence of IL on the hippocampus, they do not establish whether, during extinction, hippocampal activity is suppressed. Hippocampal suppression during extinction learning would provide strong evidence for retrieval stopping. A recent study by Winters and colleagues [120] suggests that hippocampal activity may be suppressed. Here, a single prolonged-stress model of PTSD in rodents was found to impair extinction learning and recall (processes dependent on the IL) compared to control animals. Intriguingly, the normal extinction observed in control animals was associated with reduced hippocampal metabolic activity compared to hippocampal activity in stressed animals that displayed extinction impairments. Reduced hippocampal activity in control animals was interpreted to reflect reduced fear overgeneralization. Yet, the present framework suggests a complementary interpretation: reduced hippocampal activity after extinction learning in control rats reflects successful suppression of a memory trace associated with aversive events. Paralleling this in humans, a large-scale study of PTSD in victims of the Paris terrorist attacks established that survivors who did not develop PTSD showed robust downregulation of hippocampal activity by the right DLPFC during retrieval stopping, whereas those with PTSD did not [35]. In humans, PTSD is also associated with reduced SIF [35, 121, 122], as is anxiety [123].

Although reduced hippocampal activity in Winters et al.’s could signify hippocampal inhibition in control rats, this inference is indirect. In human studies, reduced hippocampal activity during retrieval stopping has been tied to hippocampal GABA, as has suppression-induced forgetting [51]. Is there any indication that hippocampal GABA contributes to successful extinction in rodents? Although data on this topic are sparse, some evidence is supportive. For example, Yee and colleagues found that genetic disruption of hippocampal GABA A receptors containing the α5 subunit impairs fear extinction learning [124]. Thus, one important avenue to test the retrieval stopping hypothesis is to clarify the role of hippocampal GABA transmission and the specific circuitry through which it drives retrieval stopping, especially whether GABAergic action within the hippocampus relies on inputs from the nucleus reuniens.

Even if hippocampal GABA enables extinction learning, whether GABA disrupts memory for the conditioning event would remain, at best, plausible speculation. Recent findings, however, suggest that the hippocampus contains a memory of the fear conditioning experience that is suppressed by inhibition during extinction [125]. Here the authors demonstrated that acquisition vs extinction of contextual fear activated distinct ensembles of neurons (“engrams”) in the dentate gyrus of the hippocampus, as has been observed in the CA1 region [126]. They further showed that extinction was associated with reduced fear engram activation and increased activation of extinction engrams; the reverse pattern occurred during spontaneous recovery. Compellingly, selective, optogenetic manipulations of these engrams altered expression of either fear or of extinction memories. Based in part on computational analyses, Lacagnina et al. posited that increased activation of hippocampal extinction engrams actively suppresses activity of adjacent fear engrams. Although the data fit this account, it is also possible that the fear memory was not initially inhibited by the extinction engram: during extinction learning, the PFC may have directly inhibited hippocampal fear engrams via inhibitory control. The extinction engram may arise secondarily, as a biproduct of the organism’s perception of a novel event transition to a safe environment [127]. After the hippocampal extinction engram is acquired, it may assume the role of a competing trace capable of blocking the fear memory, much like thought substitution (the use of an alternative thought to block an unwanted memory) does in human studies of retrieval stopping [47]. Although these accounts remain to be tested, Lacagnina et al.’s findings vividly illustrate how extinction learning may suppress event memories for the original fear, consistent with the retrieval stopping model.

## Evidence for a role of retrieval stopping during extinction in humans

The foregoing findings suggest that during fear extinction, rodents recruit retrieval stopping processes resembling those identified in humans. Here we discuss evidence from humans that supports the retrieval stopping model. We focus on studies using fMRI or non-invasive brain stimulation, examining whether extinction engages rDLPFC and suppresses hippocampal activity.

### Right dorsolateral prefrontal cortex as a key node during extinction learning

Whereas the right DLPFC and VLPFC clearly contribute to retrieval stopping, research has rarely examined their role in fear extinction. Instead, human imaging work on extinction focuses on the ventromedial PFC, a putative human homolog of rodent IL PFC [128, 129]. However, even if VMPFC modulates amygdala activity during extinction, higher-order control regions in LPFC cortex could drive VMPFC activity to achieve emotion regulation [130]. Such a dynamic is compatible with retrieval stopping findings: effective connectivity analyses suggest that during retrieval stopping, rDLPFC negatively modulates hippocampal and amygdala activity, and retrieval stopping can reduce subjective and physiological indices of emotional reactivity to suppressed content [8, 27, 45]. If the rDLPFC and/or rVLPFC drives VMPFC, extinction learning trials should reveal activity in these regions, if people spontaneously engage in retrieval stopping.

Consistent with the foregoing proposal, human fMRI studies confirm that fear extinction engages the rLPFC. For example, in a comprehensive meta-analysis encompassing 31 studies (1074 participants), Fullana et al. [131] examined which activations consistently arose during extinction learning and recall. Most such studies involved pairing a picture (often a geometric figure) as a CS with something unpleasant (e.g., electrical stimulation to the wrist) as a US. Fullana et al. found that extinction learning engaged key regions observed during retrieval stopping, including the right aDLPFC, ACC/Pre-Supplementary Motor area, and bilateral Insula, among other regions. Fullana et al. were surprised by the DLPFC activity, because this structure is rarely discussed in individual studies. Equally surprising was that—contrary to expectation—the VMPFC region emphasized in early extinction studies was not reliably engaged. They noted, however, that the typical contrast used (comparing a CS+ to a CS− during extinction) may underestimate vmPFC engagement, insofar as, after repeated extinction trials, the CS+ would increasingly resemble the CS−. This comprehensive analysis of human studies highlights the surprising and otherwise unexplained role of rDLPFC. If right aDLPFC plays a causal role in extinction, lesions to this area should impair extinction learning, a possibility that has never been tested.

But is the rDLPFC region observed by Fullana et al. the one engaged by retrieval suppression? Although difficult to confirm without further anatomical details, other studies suggest that it might be. In a clear example, Depue et al. [5] compared, within-participants, brain regions engaged during retrieval stopping, motor response stopping, and “emotion stopping”. For retrieval stopping, Depue et al. used the Think/No-Think paradigm, with neutrally-valenced face–scene associations. For the emotion stopping task, participants viewed aversive pictures surrounded by a green or a red border. A green border signaled that participants should view the picture and fully experience the emotion conveyed (the Feel condition); a red border signaled them to “view the picture and remove themselves from any attached feeling” (Suppress condition). For motor stopping, participants performed the stop-signal task, with simple key presses. Critically, when Depue et al. examined the overlap between regions activated by all stopping tasks (action stop > go; No-Think > Think; and Suppress > Feel in the stop-signal, TNT, and emotion stopping tasks, respectively), they observed right aDLPFC activation, matching the aDLPFC cluster in Fig. 3. Thus, stopping emotional responses engages right aDLPFC, echoing work showing that keeping thoughts of physical pain out of awareness engages rDLPFC [132–134]. Although emotion stopping is not a traditional extinction task, the processes engaged plausibly are similar. Consistent with this possibility, emotion stopping reduced amygdala activity bilaterally, and diminished perceived negative valence for suppressed scenes on later ratings. When considered alongside the robust activation of right aDLPFC in the Fullana et al. meta-analysis, these findings raise the possibility that extinction engages higher-order suppressive processes to stop retrieval of thoughts about emotional and even physical discomfort elicited by conditioned stimuli.

If the DLPFC contributes to suppressing anxious thoughts, as we have proposed, then improving its functioning may alleviate anxiety and PTSD. Supporting this possibility, double-blinded randomized placebo-controlled studies verify that transcranial magnetic stimulation over the DLPFC durably reduces symptoms of anxiety and PTSD [135]. Such interventions involve repeated stimulation sessions over weeks (e.g., 20–30 sessions) to either the right or left DLPFC, using stimulation protocols ranging from low frequency (1 Hz) to higher frequency (e.g., 20 Hz) and are compared to sham stimulation. Symptom benefits are seen with right or left DLPFC stimulation, regardless of frequency; however, benefits are often greater with high-frequency stimulation over the rDLPFC, for both generalized anxiety disorder [136] and PTSD [137], with remission often persisting for months. These studies do not establish whether stimulation benefits DLPFC function or changes downstream structures to which it connects. Nevertheless, they suggest that interventions focused on rDLPFC can have lasting effects on anxiety and PTSD, consistent with a role of this structure in inhibiting unwelcome thoughts. If our retrieval stopping hypothesis is correct, stimulation interventions to the rDLPFC should improve extinction learning and its aftereffects, complementing existing work seeking to improve extinction by enhancing vmPFC activity [138].

### Hippocampal downregulation during extinction learning

RDLPFC activation during extinction learning need not imply that people are stopping retrieval of conditioning experiences. The rDLPFC simply may regulate fearful feelings elicited by the CS, without suppressing the accompanying episodic traces. To establish this possibility, one should find reduced hippocampal activity, consistent with retrieval stopping and inhibitory control over memory. Evidence consistent with hippocampal downregulation has been reported in meta-analyses of extinction learning. For example, Fullana et al. [131] reported areas less active during the CS+ (i.e., CS+<CS−). Extinction learning was associated with reduced hippocampal activity bilaterally, among other components of a broad episodic retrieval network. Additional reductions occurred in areas likely representing the fingers shocked (somatosensory cortex) and pain perception (posterior insula), all potentially contributing to suppressing the elements of the conditioning experience. Relatedly, a later meta-analysis found that people suffering from PTSD showed greater activation in the anterior hippocampus, amygdala, and posterior insula during extinction learning, compared to traumatized control participants, often arising from a failure to downregulate these structures in PTSD [139]. These human findings echo elevated hippocampal activity during extinction learning in rodent models of PTSD [140]. Failure to reduce hippocampal activity converges with evidence of deficient inhibitory regulation of hippocampal activity in PTSD observed during retrieval stopping [35]. Together, evidence from human studies of extinction learning supports the possibility that extinction engages inhibitory control over hippocampal activity, consistent with the retrieval stopping model.

## Theoretical and clinical implications

The preceding discussion illustrates how rodent and human findings conform well to predictions of the retrieval stopping model. Indeed, whereas upregulated rDLPFC and downregulated hippocampal activity follow from this model, neither follow from conventional models of extinction. Notably, although PFC and hippocampus are key to extinction, the retrieval stopping model offers a novel perspective on why this is so, and on the processes engaged. Here we elaborate on theoretical implications of these differences and their consequences for intervention.

One major implication lies in our focus on active control processes to explain extinction benefits. Most theoretical accounts conceptualize extinction as an outcome of automatic learning processes by which predictions about events are updated in response to prediction errors [141]. Thus, when an organism’s history with a CS predicts an aversive outcome, presenting the stimulus without the outcome violates a prediction. Such prediction errors might trigger reduction of the CS–US association reflecting reduced future predictions, or alternatively the storage of a novel inhibitory safety association [98, 141, 142]. Regardless of the specific learning hypothesis, however, extinction is an automatic outcome occurring without online cognitive control. Indeed, emotion regulation theorists in psychology often classify extinction as implicit emotion regulation, in which emotional changes arise without any conscious intention or involvement of cognitive control processes [143]. The retrieval stopping model assumes the opposite: that fear extinction engages cognitive control. Thus, even though emotional responding gradually reduces over extinction training without explicit instructions to stop affective responding, we argue that active stopping often (at least for mammals) occurs naturally because organisms find fear unnecessary and at odds with other pursuits. Note that the retrieval stopping process is not mutually exclusive with associative learning mechanisms. One possibility is that the capacity to stop retrieval may have evolved in mammals to work in concert with rudimentary learning mechanisms, providing greater flexibility concerning when emotional responses should or should not be expressed. Another possibility is that online, goal-directed inhibitory control arising during extinction learning may provide input that drives novel learning of inhibitory associations; once consolidated, those inhibitory associations may complement or even substitute for goal-directed control. Future work should examine how inhibitory control and associative learning contribute to major extinction phenomena and whether such mechanisms may be interdependent.

Our emphasis on active control processes raises new possibilities about the nature and timing of the mechanisms that induce change during extinction. On the one hand, associative learning approaches focus on the outcome of each trial (presence or absence of a US) as the pivotal event driving prediction error computations, yielding changes that reduce future emotion. Such approaches capture reduced affective responding over trials well but are silent about how, within an individual trial, the organism, upon realizing predictions have been violated, exits the fear state, returning to “affective baseline.” On the other hand, the retrieval stopping model focuses on regulating the current affective state, within a trial, to reduce fear judged to be unwarranted; this regulation returns the organism to a calmer state by inhibiting affective, mnemonic and even motoric representations driving behavior. Inhibition induces the immediate change in emotional state, but also, if its effects persist and accumulate, enduring effects as well. Thus, this approach captures both momentary affect regulation and trial-by-trial reductions in affective responding but is silent about how the organism infers increased safety. An ideal theoretical approach would integrate mechanisms that track and update predictions, but also account for how online regulation of affective states occurs to best explain how extinction trials reduce fear.

The retrieval stopping model also provides a novel perspective on the return of conditioned fear after extinction. One tenet of the model is that inhibitory control is engaged to stop retrieval of those aspects of an experience reactivated by retrieval cues. Given this, the inhibition of the conditioning experience need not be complete. Inhibition may be incomplete either if (a) the CS presented during extinction reactivates only a subset of conditioning event features, or (b) inhibitory control is not effectively engaged, owing to either state (e.g., stress) or trait-related deficiencies in prefrontal control. Conditioned fear should return if the cues available during later retrieval evoke features not inhibited during extinction or that provide a powerful reminder that allows a partially inhibited trace to be retrieved. Thus, during reinstatement manipulations, the US serves as a powerful reminder cue that activates enough of the original conditioning complex to renew conditioned fear when the CS appears. Even with such a reminder, however, renewal should often be imperfect; if some event features were effectively inhibited during extinction learning they should be resistant to cued retrieval by the US. Specifically, we would expect a reinstatement reduction effect, such that conditioned responding is less when a US is reinstated after extinction, compared to when a US is reinstated after a non-extinguished CS. Incomplete inhibition could also underlie spontaneous recovery, if we assume that, over time, the animal’s representation of context fluctuates so that, at retrieval, it combines with the CS to evoke more uninhibited features (cf., [144, 145]). Here again, spontaneous recovery should be incomplete, due to those features that were effectively inhibited during extinction, a phenomenon widely observed [145]. Finally, according to the retrieval stopping model, both reinstatement and spontaneous recovery should also be reduced if the same conditioning event is extinguished in response to variable stimuli, increasing the diversity of features evoked by CSs, and thus targetable by inhibition—a prediction we refer to as the suppression variability effect.

Another benefit of the retrieval stopping model lies in its potential to forge stronger connections between translational neuroscience on extinction and the human psychiatric conditions it is intended to address. An uncomfortable and not always acknowledged disconnect exists between research on extinction and disorders such as PTSD and anxiety: Whereas extinction is thought to model how experience changes fear responses to conditioned stimuli, the real symptoms faced by patients also include intrusive memories, thoughts, and images, the content of which distress the patient [146, 147]. In translating animal research on extinction to humans, researchers often either ignore the mnemonic and cognitive responses to reminders or they reconceptualize these intrusive thoughts as conditioned responses in their own right [17, 18]. Indeed, the failure of extinction research to distinguish between the threat-detection system and the cognitive representations that contribute to subjective fear led Ledoux and Pine [14] to re-evaluate the exclusive focus on the canonical extinction circuit, and to explicitly propose a two-system model of fear and anxiety, including threat-system and mnemonic components. The retrieval stopping model provides a specific framework that addresses these issues; it conceptualizes extinction not merely as reduced threat responding, but as a memory adaptation, including affective and cognitive components. This broadening of the domains of knowledge affected by extinction renders this research directly relevant to intrusive thoughts and memories, enabling it to speak to core symptoms of anxiety and PTSD.

One final implication of the retrieval stopping model concerns its consequences for clinical treatment. There is enthusiasm for the value of extinction learning as an experimental model of processes that might be deficient in clinical populations suffering from anxiety, PTSD and OCD [129, 142, 148–150]. By this view, understanding extinction mechanisms will provide evidence-based treatment targets for (a) optimizing therapies, and (b) improving extinction learning and retrieval, which may mitigate psychiatric symptoms. If the retrieval stopping model is correct, then the belief that extinction should be promoted and strengthened amounts to the belief that retrieval stopping should be promoted and strengthened. If so, practice at retrieval stopping itself may improve extinction learning. For example, practice at retrieval stopping could repeatedly present participant-designed reminders to their fears, in a standard Think/No-Think task, with instructions to suppress imagery of the feared event (see [46] for an example). If extinction on its own does not always recruit retrieval stopping, such training would increase its use and strengthen a core component process. More generally, existing therapies that build on extinction, such as exposure therapy, may already work in part because repeated exposure to feared stimuli builds skill at suppressive regulation; combining exposure therapy with training at retrieval stopping may increase its effectiveness. If these implications prove correct, they will reinforce recent challenges to the idea [9] that thought suppression is a maladaptive coping response to be avoided [151].

## Future directions

Although support for the retrieval stopping model is promising, further evidence is needed to evaluate its predictions, and to elaborate the mechanisms enabling the prefrontal cortex to regulate hippocampal activity. To encourage work on this hypothesis, we discuss areas of development in animal and human studies that could evaluate this model’s potential in translating foundational neuroscience to mental health.

One area for development in animal research is to determine whether IL PFC exerts an inhibitory influence over hippocampal activity during extinction learning, and if so, whether it suppresses neurons representing the original conditioning event. Work by Lacagnina et al. [125] shows that neurons representing the conditioning event are inhibited by the time extinction has occurred, but it remains unclear how those neurons get inhibited. An ideal study would use similar methods to track the emergence of inhibition over extinction trials and examine whether this effect is driven by IL and the neural pathways (e.g., the nucleus reuniens) hypothesized to mediate these effects. Finally, the retrieval stopping model requires evidence that extinction impairs animals’ memories for conditioning details, confirming deficient event memory, consistent with SIF.

Future research also should compare retrieval stopping and extinction in humans more precisely, using imaging and behavioral experimentation. Is the rDLPFC region engaged during extinction also involved during retrieval stopping? Moreover, although the hippocampus shows reduced activity during extinction learning, as does retrieval stopping, we do not know whether these reductions are causally related to rDLPFC activity, an issue that could be examined with effective connectivity analyses. If the hippocampus is downregulated, does this disrupt memory for the conditioning events, as retrieval stopping predicts? Translational implications can also be examined. For example, if extinction spontaneously engages retrieval stopping, can the impact of extinction be improved by instructing people to stop retrieval during extinction trials, ensuring that it always contributes (cf. [152])? To what extent do existing therapies, such as exposure therapy, capitalize on retrieval stopping, as an incidental outcome of the procedure? Together, such findings would clarify the role of retrieval stopping in extinction learning, open the door to a more complete integration between these bodies of work, and magnify their impact on mental health.

## Concluding remarks

When organisms stop actions, the result is visible to the naked eye; but when they stop thoughts or feelings, the outcome is not observable. This simple difference contributes to vastly different theoretical traditions in neuroscience relevant to stopping actions and thoughts. On one hand, the objective reality of response stopping has triggered a vast and detailed neurobiological account of how organisms stop actions, centered around constructs such as inhibitory control, which posit a dynamic, goal-related interruption of neural processes underlying action production. On the other hand, neuroscience paradigms such as extinction, are not conceptualized as stopping, even though incentives for organisms to stop memories and affect are ubiquitous. Rather, accounts of extinction focus on simple learning mechanisms applied incrementally over extinction trials. That conditioned emotional responses decrease gradually over unreinforced repetitions of a CS without special instructions or demands, in organisms simple and complex invites a basic learning account, especially given the understandable reticence to assign intentionality and control to animals. But if species such as mice and rats can engage the prefrontal cortex to stop their actions, why would they not be able to do so for affective and cognitive responses, especially when the persistence of those responses may be detrimental?

Here we built a case for the value in reconceptualizing extinction as inhibitory control over memory. We argued that extinction naturally recruits retrieval stopping to downregulate activity in the hippocampus and amygdala during extinction trials and gradually adapts mnemonic responses to conditioned stimuli. In essence, extinction reflects the organism solving a memory adaptation problem [3]. According to this retrieval stopping model, organisms are motivated to reduce aversive emotional states when circumstances do not demand them, and extinction learning communicates this change in need. When organisms detect the need to regulate emotional responses, they recruit prefrontal mechanisms to inhibit representations in the amygdala and hippocampus in parallel, reducing their influence over behavior. The cumulative effects of repeated extinction trials represent the building aftereffects of inhibitory control, and not simply associative learning. We showed that viewing human and rodent studies on extinction through the lens of retrieval stopping research reveals striking empirical parallels between the two. These parallels are sufficiently compelling to warrant further investigation. If supported, this account provides advantages over others couched solely in terms of associative learning; it would bridge extinction in non-human animals with the control of intrusive thoughts, a hallmark transdiagnostic feature of psychiatric disorders [153] and it would encourage a needed synthesis with work on inhibitory control over action and thought. Addressing these opportunities fundamentally will require a greater understanding of how the prefrontal cortex exerts inhibitory modulation over hippocampal activity [50].
